# Phenotypic effects of concomitant insensitive acetylcholinesterase (*ace-1*^*R*^) and knockdown resistance (*kdr*^*R*^) in *Anopheles gambiae*: a hindrance for insecticide resistance management for malaria vector control

**DOI:** 10.1186/s13071-014-0548-9

**Published:** 2014-12-03

**Authors:** Benoît S Assogba, Luc S Djogbénou, Jacques Saizonou, Pascal Milesi, Laurette Djossou, Innocent Djegbe, Welbeck A Oumbouke, Fabrice Chandre, Lamine Baba-Moussa, Mylene Weill, Michel Makoutodé

**Affiliations:** Institut Régional de Santé Publique, Université d’Abomey Calavi, 01BP918 Cotonou, Benin; Institut des Sciences de l’Evolution de Montpellier (UMR 5554, CNRS-UM2-IRD), Université Montpellier 2, Montpellier, France; Institut de recherche pour le développement, Unité Mixte de Recherche MIVEGEC (IRD 224-CNRS 5290-UM1-UM2), Montpellier, France; Institut de Recherche pour le Développement (IRD), MIVEGEC (UMR 224-CNRS 5290-UM1-UM2), Centre de Recherche Entomologique de Cotonou (CREC), Cotonou, Benin; Centre de Recherche Entomologique de Cotonou (CREC), Health Ministry, Cotonou, Bénin; Faculté des Sciences et Techniques, Laboratoire de Biologie et de Typage Moléculaire en Microbiologie, Université d’Abomey Calavi, 05 BP 1604 Cotonou, Benin

**Keywords:** *Anopheles gambiae*, Insecticide resistance genes, Concomitant effects, Malaria, Vector control

## Abstract

**Background:**

Malaria is endemic in sub-Saharan Africa with considerable burden for human health. Major insecticide resistance mechanisms such as *kdr*^*R*^ and *ace-1*^*R*^alleles constitute a hindrance to malaria vector control programs. *Anopheles gambiae* bearing both *kdr* and *ace-1* resistant alleles are increasingly recorded in wild populations. In order to maintain the efficacy of vector control strategies, the characterization of concomitant *kdr* and *ace-1* resistance, and their pleiotropic effects on malaria vector phenotype on insecticide efficacy are important.

**Methods:**

Larval and adult bioassays were performed with different insecticide classes used in public health following WHO standard guidelines on four laboratory *Anopheles gambiae* strains, sharing the same genetic background but harboring distinct resistance status: KISUMU with no resistance allele; ACERKIS with *ace-1*^*R*^ allele; KISKDR with *kdr*^*R*^ allele and ACERKDRKIS with both resistance alleles’ *ace-1*^*R*^ and *kdr*^*R*^.

**Results:**

Larval bioassays indicate that the homozygote resistant strain harboring both alleles (ACERKDRKIS) displayed slightly but significantly higher resistance level to various insecticides like carbamates (bendiocarb, *p* < 0.001; propoxur, *p* = 0.02) and organophosphates (chlorpyriphos-methyl, *p* = 0.002; fenitrothion, *p* < 0.001) when compared to ACERKIS strain. However, no differences were recorded between ACERKDRKIS and KISKDR resistance level against permethrin (Pyrethroid, *p* = 0.7) and DDT (Organochlorine, *p* = 0.24). For adult bioassays, the percentages of mosquitoes knocked down were significantly lower for ACERKDRKIS than for KISKDR with permethrin (*p* = 0.003) but not with deltamethrin. The percentage of mortality from adult bioassays was similar between ACERKDRKIS and ACERKIS with carbamates and organophosphates, or between ACERKDRKIS and KISKDR with pyrethroid and DDT. Concerning acetylcholinesterase enzyme, ACERKDRKIS strain showed similarAChE1 activity than that of ACERKIS.

**Conclusion:**

The presence of both *kdr*^*R*^ and *ace-1*^*R*^ alleles seems to increase the resistance levels to both carbamate and organophosphate insecticides and at operational level, may represent an important threat to malaria vector control programs in West Africa.

## Background

It is considered that 207 million cases of malaria had been the cause of 627,000 deaths annually, mainly children under five years old, in sub-Saharan Africa [1]. In general, diseases control entails prevention and treatment of human infections. However, malaria vaccines are still under experimentation and not even being used at a program level. Furthermore, malaria parasites are now showing increased resistance to anti-malaria drugs [2,3] and populations from endemic countries struggle to get access to treatments due to economic impediments [4-7].

Human malaria parasites are exclusively transmitted by *Anopheles* mosquitoes (Diptera: Culicidae). Vector control represents one of the mainstay strategies for reducing the incidence of malaria [8]. Therefore, in most of African countries, the control of mosquito vectors is the only affordable measure for the fight against malaria [9,10].

Traditional strategies aimed at tackling malaria have often focused on reducing human-mosquito contact with insecticide treated bed nets and indoor residual spraying [11-16]. However, the rapid increase in insecticide resistance in vector species is jeopardizing the successfulness of the elimination and eradication campaigns [17-22].

Insecticide Treated Nets [9] were shown to efficiently protect vulnerable populations from endemic countries [1,22,23]. Until now, pyrethroids are the only insecticide class recommended for treating mosquito nets because of their excito-repellent properties, efficacy at low-doses, and good tolerance in humans and other mammals [24]. ITNs have been used on a large scale in the last decade but pyrethroids resistance in anopheline mosquitoes were reported in all sub-Saharan Africa [25-29].

The two main mechanisms responsible for pyrethroids resistance are target site insensitivity, known as knock down resistance *kdr*^*R*^, and metabolic resistance due to elevated levels of detoxifying enzymes [21,30]. *kdr*^*R*^ resistance is caused by mutations in the sodium channel: leucine to phenylalanine substitution, originally observed in West Africa [31], and leucine to serine mutation in East Africa [32]. Recently, a new mutation in the sodium channel associated with *kdr-west* mutation conferring additional resistance to DDT and permethrin [33] was reported [34]. Experimental studies conducted in Southern Benin and in South Africa respectively with lambdacyhalothrin [33] on bednets and with deltamethrin [33] through indoor residual house spraying [35] suggested that PYRs resistance may have contributed to the failure of vector control endeavours in these areas [21,35-38].

As the main strategy for reducing malaria transmission is largely based on a limited number of insecticides [19], carbamates and organophosphates were suggested as potential alternative compounds to control pyrethroid-resistant populations [39-41]. Carbamates and organophosphates have shown a relatively good efficacy in ITNs and IRS [42-45] with high mortality of pyrethroids-resistant [46] *An. gambiae s.s* in Côte d’Ivoire [47]. However a particular concern for the use of carbamates and organophosphates is that resistance to these insecticides is already present in some *An. gambiae s.s.* populations from West Africa [29,48-53]. Carbamates and organophosphates resistance is associated with the G119S target site mutation in *ace-1* gene causing insensitivity of the AChE1 enzyme to these insecticides and to over-expression of detoxification enzyme [49,51,54,55].

In *Anopheles gambiae*, *kdr*^*R*^ and *ace-1*^*R*^ insecticide resistance alleles were found concomitantly distributed in natural populations of *An. gambiae s.s.* from West Africa [29,35,37,52,56,57]. Moreover, some individuals were found carrying both resistant alleles and *An. gambiae s.s.* populations are becoming resistant to all classes of insecticides used in vector control strategies in West Africa [29,58,59]. A synergy between *kdr*^*R*^ and *ace-1*^*R*^ alleles has been previously observed in *Culex pipiens* [46] and *An. gambiae s.s.* individuals harboring both resistance alleles could appear phenotypically more resistant to pyrethroids and carbamates/organophosphates than those harboring only *kdr*^*R*^ or *ace-1*^*R*^. Accordingly, the phenotypic effect associated with the interaction of these two resistance genes should be further investigated [29]. Moreover, *ace-1*^*R*^ resistance gene is associated with a high fitness cost in *An. gambiae* [60] and this fitness cost could be used for the development of insecticide resistance management strategies [61]. Previous studies on *Culex pipiens* showed that mosquitoes harboring both *kdr*^*R*^ and *ace-1*^*R*^ resistant alleles showed enhanced fitness compared to the one carrying only *ace-1*^*R*^ [46]. The synergy between *kdr*^*R*^ and *ace-1*^*R*^ resistant alleles could largely impede the expected success of using carbamates/organophosphates as alternative or complementary insecticides in areas where mosquitoes carry the pyrethroids resistance *kdr*^*R*^ mutation. This represents a serious threat to malaria control in the near future.

In order to sustain the efficacy of insecticide-based vector control strategies, the characterization of concomitant *kdr* and *ace-1* resistance and associated pleiotropic effects on malaria vector phenotype is relevantly important. In this study we have established a homozygote resistant strain harboring both alleles (*kdr*^*R*^ and *ace-1*^*R*^) and compared its resistance level to various insecticides to strains carrying single resistance allele.

## Methods

### Mosquito strains

Four strains of *An. gambiae* s.s. were used in this study (Table 1): i) The KISUMU reference strain, susceptible to all insecticides used in this study [62]. ii) The ACERKIS strain, which is homozygous for the G119S mutation and resistant to both OPs and CXs insecticides [51]. iii) The KISKDR strain, which is homozygous for *kdr*^*R*^ (L1014F) and confers resistance to both DDT and pyrethroids [63]. These strains have the same genetic background and metabolic resistance is not present within them. iv) The ACERKDRKIS strain was derived from the cross between ACERKIS and KISKDR strains selected with permethrin and chlorpyrifos-methyl from second to fourth generation in order to increase the frequency of individual carrying both *ace-1*^*R*^ and *kdr*^*R*^ alleles. At the fourth generation, the females blood fed were isolated in plastic cup for independently egg-laying. Their progeny were analyzed with *kdr*^*R*^ and *ace-1*^*R*^ specific molecular tests developed by Martinez-Torres *et al*. [31] and Weill *et al*. [49] respectively. When progenies displayed homozygous for *ace-1*^*R*^ and *kdr*^*R*^ alleles, they were mixed to constitute the ACERKDRKIS strain, homozygous for both resistance alleles’*ace-1*^*R*^ and *kdr*^*R*^. All strains used in this study are *An. gambiae s.s*.

**Table 1:** **Resistance of the different strains to various insecticides**

**Strains**	**Resistance mechanisms**	
	***kdr*** ^***R***^	***ace-1*** ^***R***^
Kisumu	0	0
Acerkis	0	1
KisKdr	1	0
AcerKdrKis	1	1

0 = absent; 1 = present.

*kdr* ^*R*^ = mutation associated to pyrethroids and DDT resistance.

*ace-1* ^*R*^ = mutation associated to carbamates and organophosphates resistance.

### Larval bioassay

Resistance data of the four strains (KISUMU, ACERKIS, KISKDR and ACERKDRKIS) were obtained by conducting bioassays as previously described in Djogbenou *et al*. [51].The bioassays were done in plastic cups. Late third and early fourth instars larvae were used. Six insecticides of technical grade form Sigma-Aldrich®were used: two carbamates: propoxur (99.8% pure) and bendiocarb (99.5% pure); two organophosphates: chlorpyrifos-methyl (99.9% pure), fenitrothion (95.2% pure); one pyrethroid: permethrin (98.3% pure); and one organochlorine: DDT (99.7% pure). Insecticides were diluted in 70% ethanol to make a working solution and were stored at 4°C. A set of 25 larvae was incubated in 99 ml of distilled water, to which 1 ml of insecticide solution at the required concentration was added. Four replicates were used for each concentration. Six to twelve insecticide concentrations providing a range of mortality between 0 and 100% were used for each insecticide tested. Larval mortality was recorded after 24 hours exposure. Control bioassays were conducted by adding 1 ml of ethanol to 99 ml of distilled water. Temperature was maintained at 27°C ± 2°C during bioassays test (temperature measured using Waranet kit (Waranet Solutions SAS, Auch, France)).

### WHO insecticide resistance tests on adult mosquitoes

Insecticide susceptibility tests were performed in WHO resistant test kits assays on 3–5 day old females from the four strains [64]. The tests were conducted using discriminating dosages of several insecticides used in public health as follows: two carbamates (0.4% propoxur and 0.1% bendiocarb); three organophosphates (0.4% chlorpyrifos methyl, 1.0% fenitrothion and 5% malathion); one pyrethroid (0.75% permethrin); and one organochlorine (4% DDT). Control tests were also set up by exposing adult females to untreated papers. WHO test and control papers were supplied by the WHO Collaborating Centre at University Sains Malaysia, Penang, Malaysia. Test papers were used no more than five times before being replaced. For each insecticide, six replicates were conducted. Twenty-five non blood fed females were exposed to the insecticide-impregnated test papers in the test tubes for one hour. For DDT, deltamethrin and permethrin, knockdown of females were reported every ten minutes. Mosquitoes were then transferred into holding tubes and supplied with a 10% sugar solution. Mortality was scored after 24 hours.

### AChE1 activity measurement

AChE1 activity was measured in thirty mosquitoes of each *An. gambiae ss* strain: KISUMU, ACERKIS, KISKDR and ACERKDRDKIS as described in previous studies [65,66]. Each head was individually ground and homogenized in 400 μL phosphate buffer (0.25 M, pH7) containing 1% Triton X-100. Homogenates were centrifuged (10,000 rpm for 3 min at 4°C) and 100 μL of the supernatant were used with 10 μL of ethanol (95%) for AChE1 activity measure. We then added 100 μL of 1.6 mM substrate (acetylthiocholine, Sigma, France), and AChE1 activity was estimated by measuring changes in optical density as described by Ellman et al. [67]. Colour development was measured at 412 nm for 15 min with a microplate reader Multiskan® GO and the analysis software SkanIt 3.2 (Thermo Scientific). Part of each genotype was analyzed on each plate to avoid experimental artefacts.

### Data analysis

The analyses of dose-mortality responses in bioassays were performed using the R software (v.3.0.0). The R script BioRssay (v. 5.1.1) was used; it is freely available on the website of the Institut des Sciences de l’Evolution de Montpellier [68]. This script computes the doses of insecticide killing 50% and 95% of the tested population or strain (Lethal Concentration 50 and 95, or LC_50_ and LC_95_) and the associated confidence intervals, tests for the linearity of the dose-mortality response (χ^2^ test). Finally, it allows the comparison of two or more strains or populations and calculates the resistance ratios, i.e. RR_50_ or RR_95_(= LC_50_ or LC_95_ of tested population/LC_50_ or LC_95_of the reference strain, resp.) and their 95% confidence intervals.

For adult bioassays, resistant/susceptible status was defined according to WHO criteria [1,64]. Mosquitoes were considered susceptible if the mortality rates were greater than 97% and resistant if mortality rates were less than 90%. Mortality rates between 90-97% suggested possible resistance. The knockdown times for permethrin, deltamethrin and DDT (KDT_50_ and KDT_95_) and their 95% confidence intervals were estimated using POLO-Plus (**LeOra**-**Software** 2006). The mortality difference between strains was tested using the prop.test function (based on a chi-square comparison) with the R software (v. 3.0.0).

Total acetylcholinesterase activities (*A*_*tot*_) conferred by each genotype [69] were compared using a generalized linear model as: *A*_*tot*_ *= geno + ε* where *geno* is four level factor (KISUMU, ACERKIS, KISKDR, ACERKDRKIS), ε is the error parameter following a normal distribution to take over-dispersion into account, if present. We tested significance of the different levels by Likelihood ratio tests.

## Results

### Larval bioassays

We carried out 24 larval bioassays with four replicates for the four reference strains (Tables 1 and 2). With carbamates, organophosphates, pyrethroid and DDT, the result of chi-square test between the observed dead numbers (obtained) and the dead numbers predicted by the regression log-dose probit-mortality indicated that the data were well fitted by a straight line (*p > 0.05,* Table 2) excepted for ACERKIS to propoxur (*p = 0.001*). KISUMU and KISKDR susceptibility to CXs and OPs was not significantly different (*p > 0.05*). The same susceptibility was also recorded for KISUMU and ACERKIS to pyrethroid and DDT (*p > 0.05*). These results confirm that there were no other resistance alleles involved, except the specific target site mutations (Table 1). The homozygote resistant strain harboring both alleles (ACERKDRKIS) displayed slightly but significantly higher resistance level to various carbamates (bendiocarb, *p* < 0.001; propoxur, *p* = 0.02) and organophosphates (chlorpyriphos-methyl, *p* = 0.002; fenitrothion, *p* < 0.001) when compared to ACERKIS strain (Table 2). Both ACERKDRKIS and ACERKIS displayed lower resistance level to organophosphates than to carbamates. In contrary, we did not record a significant difference between ACERKDRKIS and KISKDR resistance levels against permethrin (*p* = 0.7) and DDT (*p* = 0.24).

**Table 2:** **Log-dose and probit-mortality data for different insecticides of reference strains from** ***Anopheles gambiae s.s.***

	**Strains**										
**Insecticides**	**Kisumu**		**KisKdr**			**Acerkis**			**AcerKdrKis**		
	**LC**_**50**_ **(mg/L)**	**Chi(** ***p*** **)**	**LC**_**50**_ **(mg/L)**	**RR**_**50**_	**Chi(** ***p*** **)**	**LC**_**50**_ **(mg/L)**	**RR**_**50**_	**Chi(** ***p*** **)**	**LC**_**50**_ **(mg/L)**	**RR**_**50**_	**Chi(** ***p*** **)**
**Bendiocarb**	0,22	0,88	0.23	1	0,92	62	290	0,17	83	385	0,88
**Propoxur**	0,12	0,08	0,12	1	0,4	268	2211	<0.001	351	2899	0,42
**Chlorpyrifos-methyl**	0.004	0,8	0.004	1	0,4	0,053	12	0,33	0,065	15	0,6
**Fenitrothion**	0,004	0,13	0,004	1	0,11	0,067	16	0,77	0,11	27	0,48
**Permethrin**	0,006	0,24	0,055	9	0,89	0,006	1	0,17	0,067	11	0,26
**DDT**	0,01	0,66	0,12	12	0,34	0,01	1	0,059	0,21	21	0,47

LC_50_ is lethal concentration required to kill half of number larval tested after 24 hours.

RR_50_ is resistance ratio at LC_50_ = LC_50_(resistant strain)/LC_50_(Kisumu).

Chi(p) is indicated to judge whether the data are well fitted to the regression or not. The fits are acceptable when the p-value is over 0.05.

### Adult susceptibility

**Figure 1:**
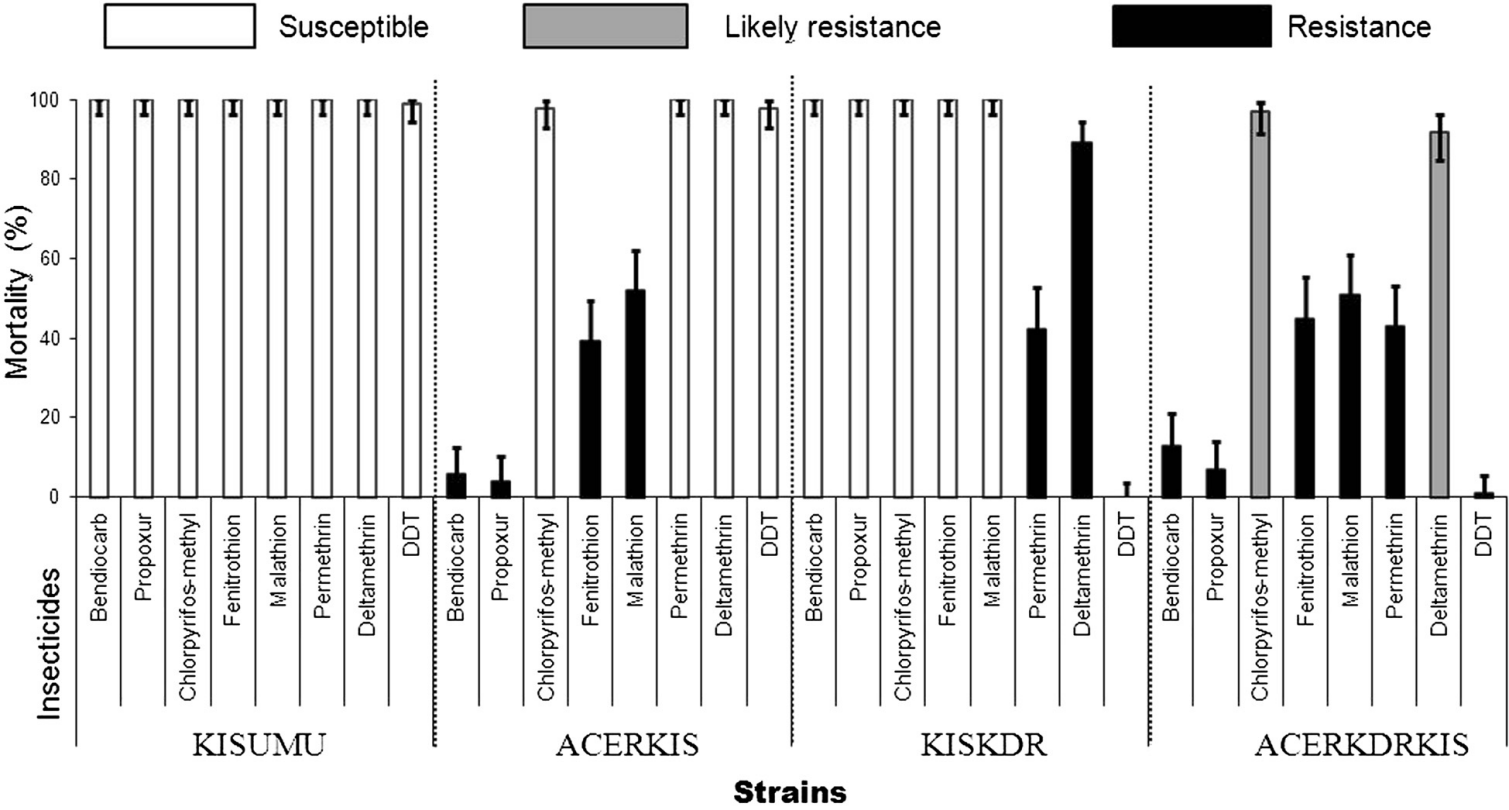
**Mortality percentages and insecticide susceptibility status of different** ***Anopheles gambiae s.s.*** **strains to insecticide.** The bars indicate the mortality percentage with 95% confidence intervals after one hour of exposure to impregnated paper in WHO test Kits and mortality reading after 24 hours.

Adult susceptibility tests with seven insecticides (propoxur, bendiocarb, malathion, chlorpyrifos methyl, fenitrothion, DDT and permethrin) were performed on the four *An. gambiae* strains. All tests used the WHO discriminating doses; 100–125 females were analyzed for each strain. Mortality in control groups was consistently *<*5%. Mortality in the susceptible strain KISUMU was above 99% for all tested insecticides. In addition, after one hour of exposure to the WHO diagnostic concentration for permethrin, the percentages of mosquitoes knocked down were significantly lower for ACERKDRKIS than KISKDR (*p* = 0.003) (Table 3). However, we did not record a significant difference between ACERKDRKIS and KISKDR knocked down percentages for deltamethrin (*p =* 1) (Table 3). DDT did not show any knockdown effect with both strains. Considering the percentage of mortality data from adult bioassays, no significant differences were recorded for bendiocarb (*p =* 0.14), propoxur (*p* = 0.56), chlorpyriphos-methyl (*p* = 0.99), fenitrothion (*p =* 0.51) and malathion (*p =* 1) between ACERKDRKIS and ACERKIS. Moreover, no significant differences were recorded between ACERKDRKIS and KISKDR mortality for permethrin (*p =* 1), deltamethrin (*p =* 0.66) and DDT (*p =* 1) (Figure 1).

**Table 3:** **Knock-down times (KdT50 and KdT95) of reference strains 1 h after exposed to insecticides**

**Insecticides**	**Strains**	**N**	**% KD**	**KdT**_**50**_	**CI**_**95**_	**KdT**_**95**_	**CI**_**95**_	**KdT**_**50**_ **R**
**Permethrin**	Kisumu	98	100	8.6	7.6-9.6	28.5	24.7-34	-
	Acerkis	100	100	10.9	9.5-12.4	25.2	21-32	1,3
	KisKDR	99	23,23	178	114-414.5	2544	864-21000	20,6
	AcerKdrkis	100	7	No KdT	-	No KdT-	-	-
**Deltamethrin**	Kisumu	101	100	7.7	6.8-8.5	21	18.3-25	-
	Acerkis	96	100	8	7.2-8.7	17.8	15.4-21	1
	KisKDR	101	94	19.3	15-23.5	51	39.6-77.4	2,5
	AcerKdrkis	100	94	27	24-30	55.7	48-69.4	3,5
**DDT**	Kisumu	99	98	33.6	31-36	50.1	45.6-57.7	-
	Acerkis	100	77	45	43-47	77.5	70.7-88	1.3
	KisKDR	98	0	No KdT	-	No KdT	-	-
	AcerKdrkis	100	0	No KdT	-	No KdT	-	-

N = number of mosquitoes tested.

KdT_50_R, KdT50 of the tested population divided by KdT50 of the Kisumu (susceptible reference strain).

% KD = percentage of mosquitoes knock-down after 60 mn.

KdT_50/95_ = Knock-down times in minutes for 50 or 95% of adult mosquitoes after one hour of exposure to impregnated paper in WHO test Kits.

CI _95_ = 95% confidence intervals.

No KdT = Complete loss of KD effect (less than 5% of KD mosquitoes after one hour exposure).

### Total AChE1 activity

**Figure 2:**
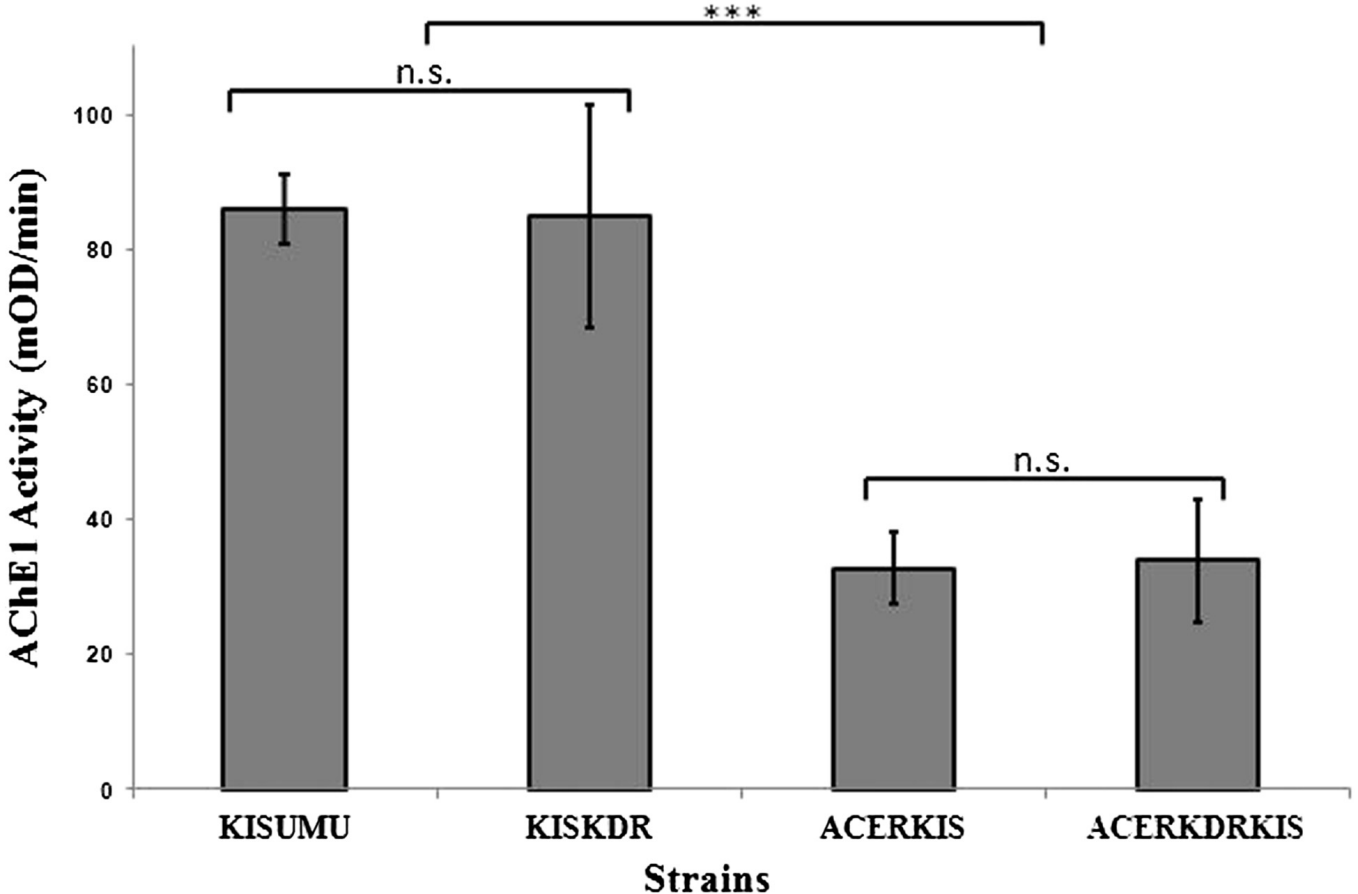
**Total AChE1 activity for females of different** ***Anopheles gambiae*** **s.s. strains.** The bar indicate the mean AChE1 activity in mosquito head from susceptible and resistant *Anopheles gambiae* s.s. strains (KISUMU (N = 30), KISKDR (N = 30), ACERKIS (N = 30), and ACERKDRKIS (N = 30). ***indicated significant difference (p < 0.001), n.s. indicated not significant difference (p > 0.05).

The total AChE1 activity was measured for thirty individuals of each reference strains. We found a significant reduction of AChE1 activity for ACERKIS and ACERKDRKIS strains in comparison to KISUMU and KISKDR strains (*p* < 0.001, Figure 2). However the AChE1 activity for ACERKDRKIS (34 ± 9mOD/min) and ACERKIS (33 ± 5mOD/min) strains was not significantly different (*p* = 1) and KISKDR (85 ± 16mOD/min) strain displayed similar AChE1 activity as KISUMU (86 ± 5mOD/min) strain (*p* = 1).

## Discussion

The occurrence of multiple resistance mechanisms towards insecticides in single specimen of *An. gambiae* has been shown in several studies in West Africa [29,52]. In this study, we aimed at characterizing the impact of concomitant occurrence of *kdr*^*R*^ (L1014F) and *ace-1*^*R*^ (G119S) resistance alleles in single *An. gambiae s.s.* by establishing the ACERKDRKIS strain, which harbor the *kdr*^*R*^ and *ace-1*^*R*^ resistance alleles.

ACERKDRKIS strain displayed slightly but significant higher resistance levels to various carbamates and organophosphates when compared to ACERKIS strain. These observations were also shown in *Culex quinquefasciatus* where the BSCR strain harboring both alleles displayed a resistance level to carbosulfan (carbamates) higher than that of the SR strain (*Culex quinquefasciatus* homozygotes for the resistance allele *ace-1*^*R*^) [46]. These results suggest that the presence of *kdr*^*R*^ mutation in individuals harboring *ace-1*^*R*^ allele may contribute to the higher resistance levels observed against carbamates and organophosphates in *An. gambiae s.s.* We cannot clearly explain this data, although when the original targets of insecticides become insensitive and higher doses are required to achieve equivalent mortality in the strains harboring both insensitive targets of insecticides, secondary target sites may be involved [70-72].

Our study did not detect a significant difference for pyrethroids and DDT mortality rate between ACERKDRKIS and KISKDR in larval and adult stage suggesting that *ace-1*^*R*^ mutation does not influence mosquito resistance to pyrethroids and DDT. However, our results detect a significant reduction of knockdown percentage for permethrin but not for deltamethrin (Table 3). This may depend on the types of pyrethroids compound because permethrin and deltamethrin are respectively from type I and II of pyrethroids, and type II have greater toxicity than type I [73-77]. However, this knockdown reduction in *ace-1*^*R*^ and *kdr*^*R*^ concomitant distribution area, may affect the malaria transmission risk in community.

We found a significant reduction of AChE1 activity from *An. gambiae* homozygous for *ace-1*^*R*^ (resistant strain ACERKIS) in comparison to KISUMU, the susceptible control strain (Figure 2). Same results were observed in previous studies focused on comparison of *An. gambiae* and *Culex pipiens* acetylcholinesterase 1 biochemical properties [65]. This activity reduction of insensitive AChE1-R may be responsible for fitness cost associated with *ace-1*^*R*^ mutation [60,65]. We did not find a significant difference between AChE1 activity of ACERKDRKIS individuals harboring both *ace-1*^*R*^ and *Kdr*^*R*^ compared with ACERKIS individuals (homozygous for *ace-1*^*R*^) (Figure 2). Our findings showed as expected that *kdr*^*R*^ mutation does not interact with AChE1 activity. Thus, if the fitness of double mutants is improved as it was showed for *Culex pipiens* [46], it will not be through the increase of AChE1 activity. Further studies on fitness cost in free insecticide environment should be conducted on *An. gambiae* strains harboring only one resistance allele (either *ace-1*^*R*^ or *kdr*^*R*^), both alleles, or no resistance allele to answer this question.

## Conclusion

This study demonstrated that *ace-1*^*R*^ and *kdr*^*R*^ alleles interact to enhance resistance to carbamates and organophosphates. These results show that the concomitant occurrence of acetylcholinesterase (*ace-1*^*R*^) and knockdown resistance (*kdr*^*R*^) in *An. gambiae* could be a great concern for carbamates and organophosphates use as alternatives against pyrethroids resistance. The cost reduction in *An. gambiae* double resistant could facilitate the spread of these targets resistance mechanisms in natural populations of vector. This represents a major threat for insecticide resistance management for malaria vector control. The results of this study should be carefully considered while elaborating malaria vector control programs in West Africa.
